# Case Report: *Aspergillus fumigatus*-associated plastic bronchitis in a pediatric patient with cystic fibrosis complicated by allergic bronchopulmonary aspergillosis

**DOI:** 10.3389/fped.2025.1632989

**Published:** 2025-08-26

**Authors:** Fen Liu, Linlin Zhao, Yanpeng Gao, Xiaoyan Wang, Han Zhang

**Affiliations:** ^1^ Department of Pediatrics, Shengjing Hospital of China Medical University, Shenyang, China; ^2^ Department of Pediatrics, The First Hospital of Yulin, Yulin, China

**Keywords:** plastic bronchitis, bronchial casts, *Aspergillus fumigatus*, fiberoptic bronchoscopy, allergic bronchopulmonary aspergillosis, cystic fibrosis

## Abstract

While plastic bronchitis (PB) is most commonly associated with viral pathogens and *Mycoplasma pneumoniae* (MP) infections, fungal etiologies are exceptionally uncommon in children. We described a case of a 10-year-old girl with *Aspergillus fumigatus*-induced PB. She had a 10-month history of intermittent coughing as the predominant symptom, without acute progressive dyspnea. Her medical history included cystic fibrosis (CF) complicated by allergic bronchopulmonary aspergillosis (ABPA). The bronchoscopy revealed a large amount of thick, gelatinous mucus plugs in the right lower lobe bronchus. After two fiberoptic bronchoscopy procedures, the plugs were removed and turned out to be large gelatinous bronchial casts. Fungal culture of the bronchoalveolar lavage fluid (BALF) was positive for *Aspergillus fumigatus*, and pathological examination of the bronchial casts revealed *Aspergillus* hyphae. This case demonstrates a chronic onset process, with significantly delayed cast formation compared to PB cases caused by other pathogens. Notably, *Aspergillus fumigatus*-associated casts exhibited marked differences in CT imaging characteristics, gross morphological features and histopathological profiles when compared to MP-associated casts. The current case suggests that *Aspergillus fumigatus* may be an additional cause of PB. Clinicians should include this pathogen in the differential diagnosis of patients presenting with prolonged symptom duration, CT evidence of high-attenuation mucus plugs and casts exhibiting gelatinous texture.

## 1 Introduction

Plastic bronchitis (PB) is a disease characterized by endogenously formed foreign bodies that cause bronchial obstruction, leading to impaired pulmonary ventilation function (1). Bronchial casts form due to increased mucin glycoprotein secretion in response to interactions between airway epithelial cells and external antigens. These casts are composed of a mixture of mucin glycoproteins, water, ions, proteins, and lipids. They have an abnormally thick consistency, are semi-solid in form, and take the shape of the bronchial tree when removed. The casts can partially or extensively obstruct the airways, resulting in clinical symptoms such as chest tightness, shortness of breath, respiratory distress, and even respiratory failure, which can be life-threatening in severe cases (2, 3). Prompt recognition of this disease and the removal of the casts are crucial.

PB has historically been considered rare in both adults and children. However, with the increasing use of bronchoscopy in pediatric cases, PB in children has become more widely recognized (4, 5). The causes of PB are diverse and can be infectious or non-infectious. Among infectious causes of PB in children, the most commonly reported pathogens include *Mycoplasma pneumoniae* (MP) and respiratory viruses (4, 6, 7). Current evidence suggests fungal etiologies represent an uncommon cause of plastic bronchitis, with limited cases documented to date (8).

This article presents a case of PB caused by *Aspergillus fumigatus* infection. It discusses the case from multiple perspectives, including clinical symptoms, imaging findings, bronchoscopic appearance, pathology of the casts, and characteristics of bronchoalveolar lavage fluid. By reviewing the literature, we aim to highlight the unique features of this case and differentiate PB caused by *Aspergillus fumigatus* from PB due to other infectious pathogens. The goal is to familiarize pediatricians with the characteristics of infectious PB caused by different pathogens. This study may enhance early recognition of *Aspergillus fumigatus*-induced PB based on symptoms duration, CT imaging, and bronchoscopic findings.

## 2 Case presentation

**Figure 1:**
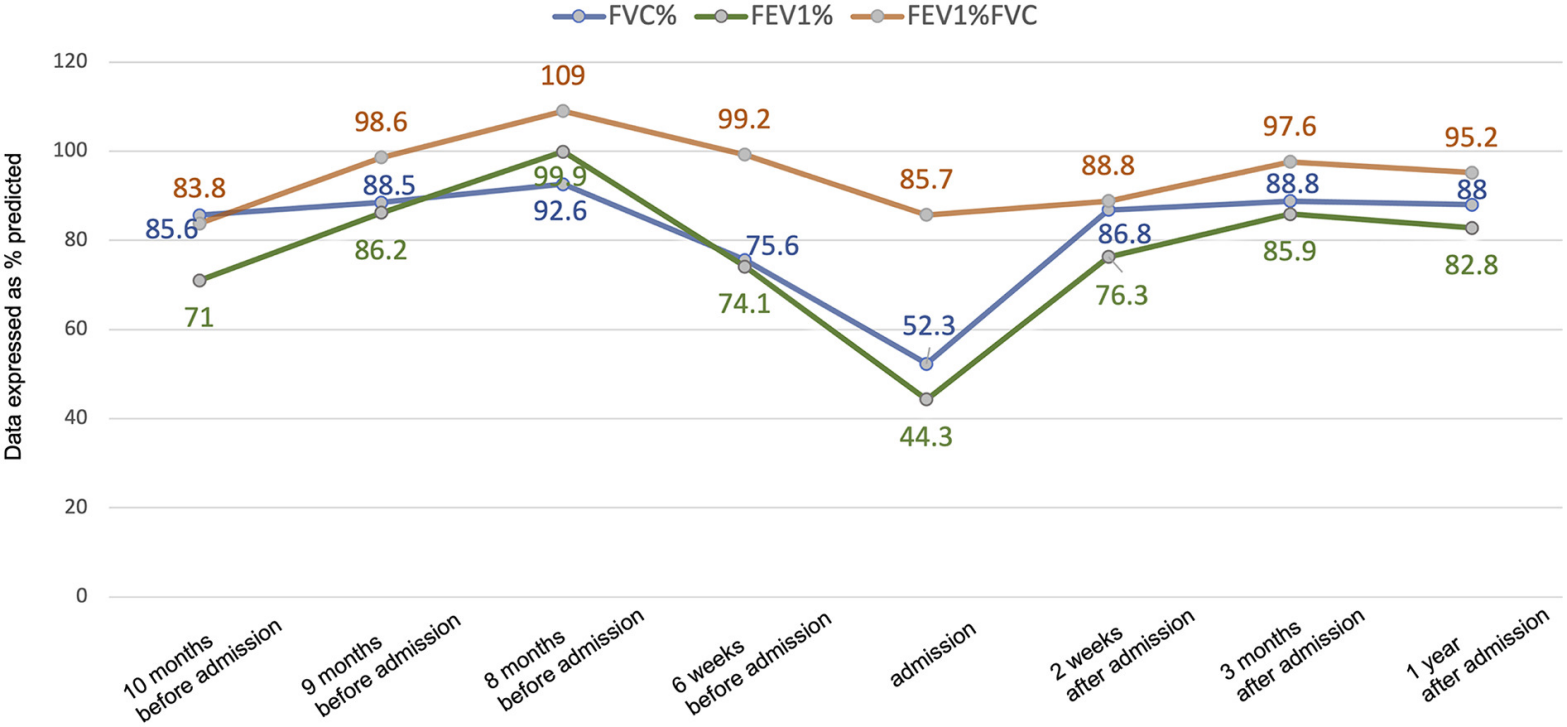
Changes in pulmonary function before and after treatment. Lung function data are presented as % predicted. FVC, forced vital capacity; FEV1, forced expiratory volume in 1 s.

A 10-year-old girl was admitted due to intermittent cough for 10 months. The patient was diagnosed with cystic fibrosis (CF) at age of 3 following identification of a heterozygous mutation in the cystic fibrosis transmembrane conductance regulator (*CFTR*) gene. Ten months prior to this admission, the patient had been hospitalized for cough and hypoxia. Chest computed tomography (CT) revealed bilateral bronchiectasis and pulmonary infection. Pulmonary function tests indicated mild obstructive ventilatory dysfunction, and the bronchodilator test was positive (Figure 1). Fiberoptic bronchoscopy revealed mild dilation at the opening of the B6, B8, B9, and B10 bronchial segments in the left lower lobe, and copious yellow-white viscous secretions were observed in the bronchi of both lungs. Bronchoalveolar lavage fluid (BALF) culture revealed Pseudomonas aeruginosa infection, and fungal spores were found in the BALF fungal smear. She received IV ceftazidime (1 g q12 h) and voriconazole (6 mg/kg q12 h loading dose then 4 mg/kg q12 h maintenance) for 10 days. After treatment, the cough was alleviated and the hypoxia was corrected. Post-discharge, the patient continued oral levofloxacin (8 mg/kg q12 h) for 2 weeks until follow-up sputum cultures turned negative. She continued voriconazole (4 mg/kg q12 h) for 6 months orally. For long-term control of allergic bronchopulmonary aspergillosis (ABPA), inhaled salmeterol/fluticasone combination (50/100 μg per actuation) was administered via dry powder inhaler twice daily over 6 months. After discharge, the patient had intermittent coughing without sputum or wheezing. Two follow-up results showed normal pulmonary function and negative bronchodilation test (Figure 1). Six weeks earlier, the patient experienced fever for 2 days, followed by a productive cough without wheezing or dyspnea. The sputum bacterial and fungal cultures were negative. She received oral levofloxacin (8 mg/kg q12 h) for 7 days. However, 3 days earlier, her cough worsened, severely affecting her sleep, prompting another hospitalization.

**Figure 2:**
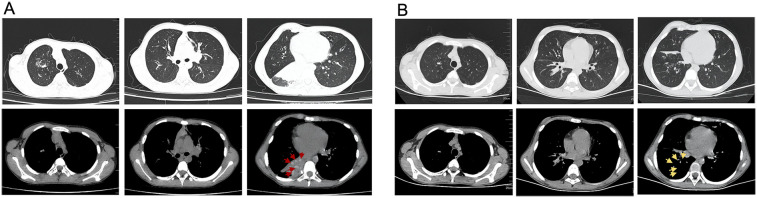
Changes on chest CT before and after treatment. **(A)** Chest CT scans showing right lower lobe atelectasis and high-density shadows in the bronchus of right lower lobe before treatment. The red arrows indicates the casts with a CT attenuation value of 81 HU in the mediastinal window. **(B)** Chest CT scans showing re-expansion of the right lower lobe after treatment. Post-clearance CT changes are demarcated by yellow arrows.

Upon this admission, physical examination revealed: temperature 36.5°C, pulse 122 beats per minute, respiratory rate 36 breaths per minute, and oxygen saturation of 91% without supplemental oxygen. The patient was alert, with no abnormalities in skin or mucous membranes, no nasal flaring or intercostal retractions. Lung auscultation showed slightly diminished breath sounds without dry or wet rales. Chest CT showed pulmonary infection in both lungs, high-attenuation shadows in the right lower lobe bronchus, right lower lobe atelectasis, a small amount of pleural effusion on the right side, and bilateral bronchiectasis (Figure 2A). Pulmonary function tests revealed moderate mixed ventilatory dysfunction (Figure 1), with a positive bronchodilator response. Blood tests showed: white blood cells 9.31 × 10^9^/L, neutrophils 53.3%, C-reactive protein 2.8 mg/L. Total IgE was 4,819 KUA/L, with strong positive results for *Aspergillus fumigatus* specific IgE, mixed fungal specific IgE, and Alternaria specific IgE. Serum galactomannan (GM) and (1,3)-β-D-glucan (G) tests were negative, as were respiratory viral nucleic acid tests for influenza virus, adenovirus, MP, and respiratory syncytial virus. Sputum bacterial cultures were negative.

**Figure 3:**
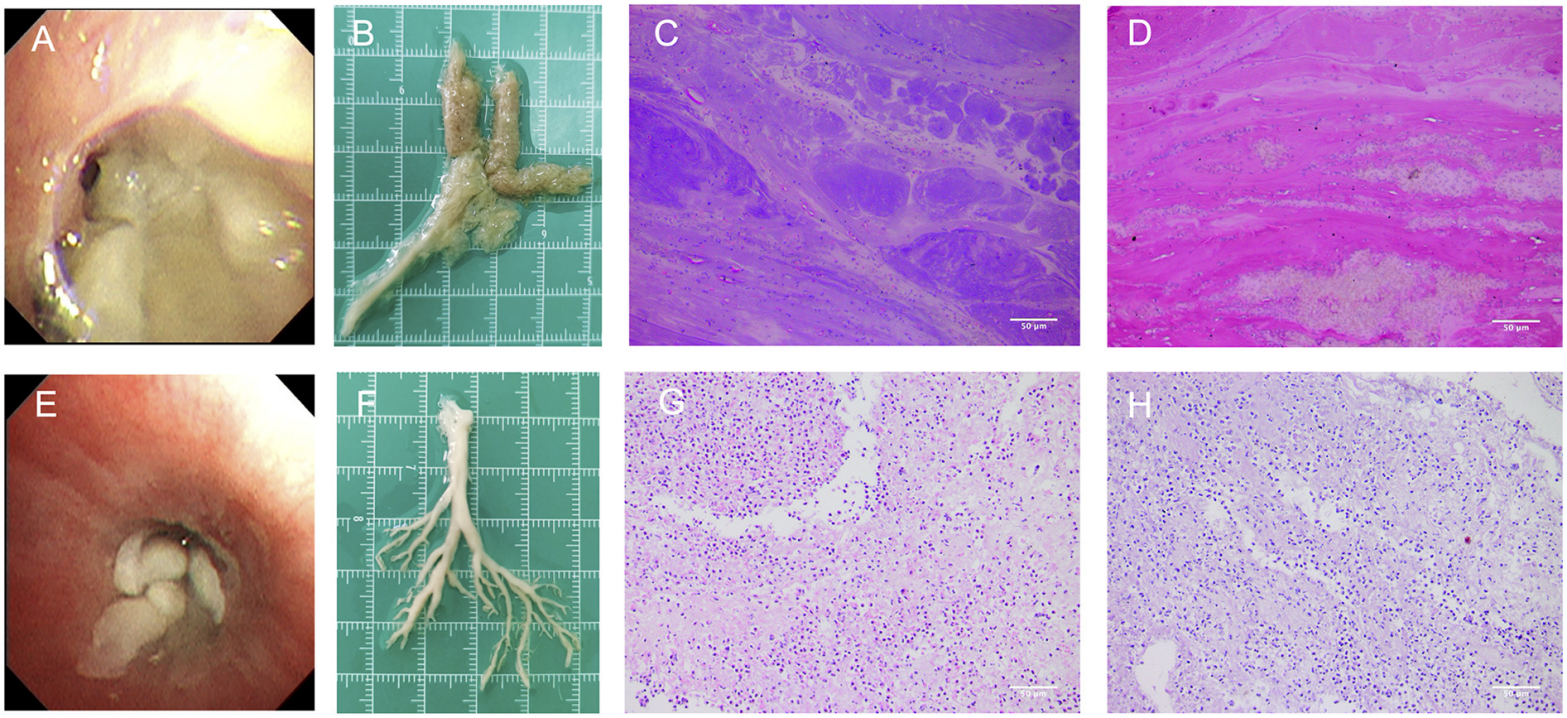
Gross morphology and histopathological findings of bronchial casts. **(A)** The appearance of *Aspergillus fumigatus* casts under bronchoscopy. **(B)** *Aspergillus fumigatus*-induced casts had a rough surface, loose internal structure, and a gelatinous texture. **(C,D)** HE and PAS staining of *Aspergillus fumigatus* casts showed significantly more mucus and appeared more alkaline (light microscopy, magnification ×200). **(E)** The appearance of MP casts under bronchoscopy. **(F)** MP-induced PB sample from another patient had a smooth surface, denser internal structure, and a tougher texture. **(G,H)** HE and PAS staining of MP-induced casts showed higher inflammatory cell infiltration and more fibrin than mucus (light microscopy, magnification ×200).

**Figure 4:**
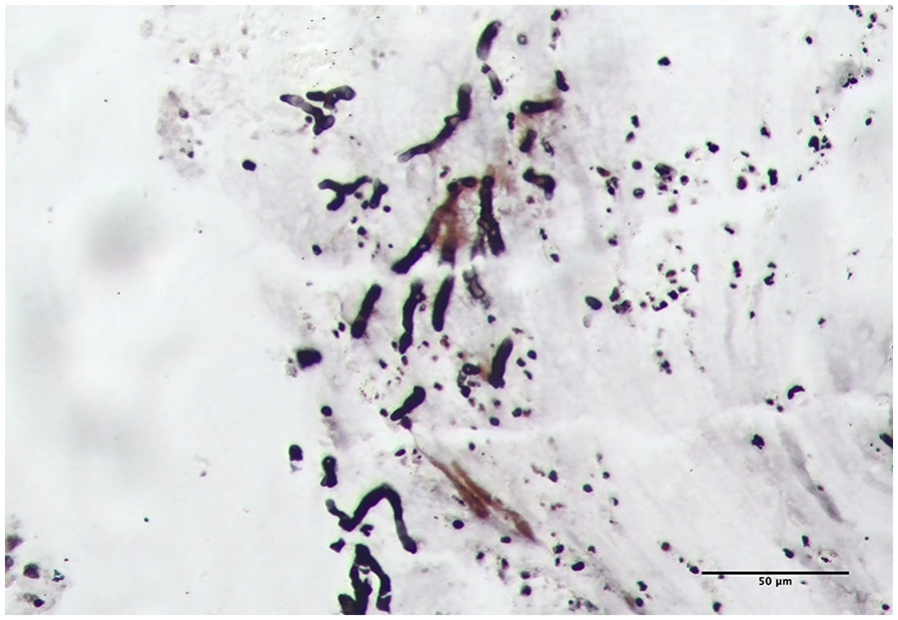
*Aspergillus* hyphae were identified on Gomori methenamine silver staining of the casts (light microscopy, magnification ×200).

Upon admission, the patient received oxygen via face mask at 5 L/min, with oxygen saturation increasing to over 97%. On the second day of admission, bronchoscopy revealed yellow-white dense secretions obstructing the right lower lobe bronchial openings (B6, B7, B8, B9, B10) (Figure 3A) and the left lower lobe (B8), which were difficult to aspirate. Two bronchoscopies were performed on the second and fourth days of admission. During these procedures, large gelatinous plastic mucus plugs were removed using biopsy forceps and brushes, which turn out to be bronchial casts, approximately 6 cm by 5 cm in size (Figure 3B). After the lavage, the bronchial openings in the left and right lower lobe were clear. The pathology of the gelatinous casts revealed a large mucin component with neutrophil-dominant inflammatory cell infiltration. Periodic Acid-Schiff (PAS) staining was positive (Figures 3C,D). Gomori methenamine silver staining demonstrated characteristic dichotomously branching, septate hyphae morphologically consistent with Aspergillus species within the cast material (Figure 4). Fungal culture from the lavage fluid confirmed Aspergillus fumigatus growth. Cell count of BALF: neutrophils 70%, macrophages 15%, lymphocytes 14%, epithelial cells 1%. After the second bronchoscopy the patient's oxygen saturation improved to normal.

During hospitalization, the diagnosis of ABPA was established (cystic fibrosis, Aspergillus fumigatus sensitization, elevated total IgE >1,000 IU/ml, and central bronchiectasis). Fungal elements observed within the bronchial cast (Figure 4) represent airway colonization consistent with ABPA pathophysiology. The patient received a comprehensive therapeutic regimen targeting distinct pathological processes: Intravenous methylprednisolone (1 mg/kg/day for 11 days) was administered to control ABPA-related airway inflammation, while antifungal therapy consisted of an IV voriconazole (6 mg/kg q12 h loading dose then 4 mg/kg q12 h maintenance) for 7 days and subsequent oral stepdown (4 mg/kg q12 h) for 4 days. Concurrent antibacterial coverage against *Pseudomonas* was provided by IV ceftazidime (1 g q12 h) for 11 days, complemented by mucolytic therapy with nebulized acetylcysteine (3 ml q12 h) for 11 days to facilitate bronchial cast dissolution. Her cough and sputum production significantly improved, and follow-up CT showed re-expansion of the right lower lobe, resolution of pleural effusion, and unchanged bronchiectasis (Figure 2B). The patient was discharged.

Post-bronchoscopy, her pulmonary function significantly improved from moderate obstructive dysfunction to mild obstructive dysfunction: forced vital capacity (FVC) increased from 52.3% to 86.8%, forced expiratory volume in 1 s (FEV1) increased from 44.3% to 76.3%, and peak expiratory flow (PEF) increased from 46.8% to 65.5% (Figure 1). Following discharge, the patient continued maintenance therapy comprising: oral prednisone initiated at 0.5 mg/kg/day and tapered over 3 months; oral voriconazole (4 mg/kg q12 h) and inhaled salmeterol/fluticasone combination therapy (50/100 μg per dose via metered-dose inhaler twice daily) for 3 months. Outpatient follow-up at 3 months and 1 year after discharge showed normal pulmonary function (Figure 1).

## 3 Discussion

PB is a rare respiratory disorder characterized primarily by the formation of plastic mucus plugs within the airways. This disease typically presents acutely, with airway obstruction caused by the plugs, leading to symptoms of respiratory distress and respiratory failure (2, 9, 10). As a potentially life-threatening respiratory condition, PB exhibits heterogeneous etiologies. The classification proposed by Seear et al. stratifies PB into type I and Type II (1). Type I, the inflammatory or cellular type, is caused by airway obstruction and hypoxia, which usually has an acute onset and is commonly seen in patients with respiratory diseases such as asthma, bronchopneumonia, and atelectasis (11). Type II, the non-inflammatory or acellular type, is typically caused by pulmonary venous hypertension, heart failure, abnormal pulmonary blood flow or lymphatic return obstruction (12, 13). In this case, the absence of halo signs, cavitations, ground-glass opacities, or centrilobular nodules on CT distinguished this presentation from invasive pulmonary aspergillosis and hypersensitivity pneumonitis. Bronchoscopic findings ruled out foreign body aspiration. Definitive pathological examination of the cast excluded pulmonary tuberculosis and neoplastic airway diseases. This case represents *Aspergillus fumigatus*-associated plastic bronchitis in a patient with cystic fibrosis complicated by allergic bronchopulmonary aspergillosis. However, the onset, CT findings, appearance and pathology of the plastic casts were notably different from previously reported cases of Type I PB. Therefore, this article discusses these aspects in relation to the existing literature to deepen our understanding of the disease and assist in the early recognition of fungal-induced PB.

This case presented with a prolonged clinical course compared to most infection-associated PB cases, with a cough lasting for 10 months. Although there is no consensus exists regarding the typical duration for cast formation, existing literature reveals pathogen-dependent temporal patterns in PB.

Influenza-induced PB exhibits rapid progression (10, 14–16). Three confirmed Influenza A virus H1 cases demonstrated respiratory distress within 24 h of symptom initiation, with bronchoscopic confirmation of casts by day 2 (10, 15). A parallel case involving influenza B virus showed PB onset within 72 h (16). Hu et al. reported 36 pediatric PB cases caused by influenza A or B viruses, with a median fever duration of 8 days (14). Adenoviral PB may follows distinct temporal patterns (17–19). Two documented cases revealed cast formation latency, with detection at days 10 and 21 post-infection respectively (18, 19). In cases of severe adenovirus pneumonia with PB, the median fever duration was 12.5 days (17). Respiratory syncytial virus (RSV) and human bocavirus (HBoV) associated PB are relatively rare. They tend to have a more abrupt symptom onset and severe respiratory compromise. An immunocompromised child with RSV-induce PB developed progressive respiratory failure (day 4), pneumomediastinum (day 11), and required extracorporeal membrane oxygenation (ECMO) by day 14 (20). A previously healthy 3-year-old boy with HBoV-induced PB deteriorated despite mechanical ventilation, requiring veno-venous ECMO within 24 h of admission (9). Another 20-month-old boy with HBoV-induced PB developed respiratory distress and atelectasis 3 days after fever onset, progressing to mechanical ventilation on day 4 (6).

MP-associated PB has emerged as a predominant etiology of PB in school-aged children in Asia (21, 22), demonstrating similar temporal pattern with adenoviral PB. In our previous study of 52 children with MP-associated PB, the mean time of cast formation was 10.44 days (4). Another study reported 13 out of 15 pediatric MP-associated PB cases, with a time of cast formation ranging from 3 to 15 days (7). Yang et al. studied 133 cases of PB associated with MP, reporting a median time from illness onset to admission of 7 days (21). MP-associated PB is more commonly presented with prolonged fever durations, and casts typically appearing within 2 weeks of illness onset (4, 21, 22). Emerging evidence implicates bacterial pathogens in PB, though temporal progression patterns remain poorly known. A *Bordetella parapertussis*-associated PB case showed cast detection at day 7 post-onset (23), while *Haemophilus influenzae*, *Streptococcus pneumoniae*, and *Moraxella catarrhalis* have been implicated without documented temporal progression (5, 24).

Reports of PB caused by fungal infections remain relatively uncommon in the medical literature, particularly in children (25). In this case, the girl had an unusually prolonged disease course without acute respiratory distress, which was distinct from the PB cases described above. We hypothesize that high levels of *Aspergillus fumigatus* exposure led to airway inflammation and excessive secretion production. Additionally, the patient's *CFTR* gene mutation resulted in impaired ion transport and reduced mucociliary clearance, leading to gradual mucus accumulation, airway obstruction, and subsequent ventilation impairment with hypoxia (11, 26). This slow accumulation of secretions over months likely accounts for the absence of acute respiratory distress. In summary, *Aspergillus fumigatus* infection and the underlying CF condition were key factors contributing to the chronic process of cast formation in this patient.

Beyond differences in cast formation, this case also exhibited unique CT findings. Abnormally high-attenuation mucus (HAM) plugs were observed around the right lung hilum, with a CT attenuation value of 81 HU in the mediastinal window—significantly higher than the adjacent atelectatic lung tissue (45 HU) and even higher than the paraspinal muscle density (60–70 HU) (Figure 2A). In contrast, PB caused by MP typically has mucus densities closer to normal lung tissue. The mechanism underlying HAM formation remains unclear. Some studies suggest that mucus density is related to protein concentration within the mucus (27). Normal tracheal mucus consists of 95%–98% water and 2%–5% solids, primarily glycoproteins. Because of its high water content, mucus typically appears with a density close to water on CT. However, in some pulmonary neoplastic diseases, mucus accumulation occurs slowly, leading to increased mucus protein secretion or water reabsorption, which raises the CT attenuation value to above 28 HU, sometimes reaching as high as 130 HU (27). Other studies suggest that increased mucus density is associated with calcium and metal salt deposition (28–30). We speculate that high-attenuation casts may be a distinctive feature of fungal PB, with potential diagnostic value in differentiating PB etiology.

Additionally, the plastic casts differed in gross morphology and pathology compared to typical PB cases. The *Aspergillus fumigatus* induced casts had a rough surface, loose internal structure, and a gelatinous texture, making them slippery and difficult to grasp with biopsy forceps (Figures 3A,B). In contrast, a MP-induced PB sample from another patient had a smooth surface, denser internal structure, and a tougher texture, making it easier to grasp with forceps (Figures 3E,F). Histopathological analysis revealed that both casts exhibited predominant neutrophil infiltration with some eosinophils and histiocytes (Figures 3C,G). However, HE and PAS staining showed that *Aspergillus fumigatus*-induced casts contained significantly more mucus and appeared more alkaline, whereas MP casts had higher inflammatory cell infiltration and more fibrin than mucus (Figures 3C–H). In ABPA, airway mucus plugging is a well-documented phenomenon. However, the present case represents a distinct entity. Unlike the amorphous mucus plugs characteristic of ABPA, the bronchial cast observed here demonstrated preserved branching architecture mirroring the bronchial tree. Moreover, while ABPA-related plugs typically contain numerous eosinophils and Charcot-Leyden crystals, histopathological examination of this cast revealed predominant neutrophil infiltration with embedded Aspergillus hyphae—features inconsistent with classic ABPA pathology (11).

Currently, there is no standardized guideline for the treatment of pediatric PB. The widely accepted approach is early removal of the bronchial casts using fiberoptic bronchoscopy. Bronchoscopy plays a crucial role in both diagnosing and treating PB by rapidly clearing endogenous obstructions, improving lung ventilation, and quickly alleviating respiratory distress symptoms (7). Additionally, bronchoscopy allows for the direct administration of medications into the distal airways for enhanced therapeutic effects. Other treatment modalities for PB include inhalation of 3% hypertonic saline, bronchodilators, tissue plasminogen activator, acetylcysteine, and nebulized heparin inhalation (31–34). Furthermore, since different underlying diseases lead to varying mechanisms and severities of cast formation, treatment should be tailored accordingly. This may involve intravenous antibiotics, corticosteroids, and respiratory support therapy (4, 20, 35). There have also been reports of using mepolizumab injections and inhaled interferon for treatment (18, 36). In this case, the patient underwent two fiberoptic bronchoscopy procedures on days 2 and 4 of hospitalization. By day 5, oxygen saturation had returned to normal, indicating that airway obstruction caused by the casts had been resolved. Additionally, treatment targeted the underlying condition of ABPA, with voriconazole administered for antifungal therapy, methylprednisolone used to suppress inflammation, inhaled acetylcysteine to break down thick mucus, and prophylactic ceftazidime to prevent potential Gram-negative bacterial infections. A follow-up chest CT on day 8 showed re-expansion of the right lower lobe, reduced inflammation in both lungs, and absorption of pleural effusion, demonstrating a positive treatment outcome. Taken together, the management of *Aspergillus fumigatus*-induced PB should prioritize immediate relief of respiratory obstruction as the primary intervention, followed by targeted antifungal therapy with appropriate course duration and dosage. Adjunctive symptomatic therapies should be concurrently implemented to optimize clinical outcomes.

In conclusion we present a novel case of *Aspergillus fumigatus*-associated PB characterized by chronic progression, high-attenuation mucus impactions on CT, rough-surfaced and gelatinous bronchial casts, and histopathological dominance of basophilic mucoid components. The current case expands the microbial spectrum of PB, establishing *Aspergillus fumigatus* as a potential pathogen in children with PB. Fungal PB should be considered in cases demonstrating persistent symptoms, particularly when accompanied by HAM plugs on imaging. Understanding these characteristics can help pediatricians make more informed clinical decisions.

## Data Availability

The original contributions presented in the study are included in the article/Supplementary Material, further inquiries can be directed to the corresponding author.
